# Differences in Public Search Interest for Anterior Cruciate Ligament and Medial Collateral Ligament Injuries: A Google Trends Analysis

**DOI:** 10.7759/cureus.112677

**Published:** 2026-07-14

**Authors:** Nikhil Maddina, Aditya J Bhatt, Yincheng Jin, Mit Patel, Saad Siddiqui

**Affiliations:** 1 Medicine, Midwestern University Chicago College of Osteopathic Medicine, Downers Grove, USA; 2 Orthopedic Surgery, HCA (Hospital Corporation of America) Florida JFK Hospital, Atlantis, USA

**Keywords:** anterior cruciate ligament (acl), health care literacy, medial collateral ligament injury, orthopedic sports medicine, patient education, readability score

## Abstract

Introduction: Anterior cruciate ligament (ACL) and medial collateral ligament (MCL) injuries are commonly encountered injuries in orthopedic practice. Simultaneously, patients are utilizing online resources to better understand diagnoses and treatment options. As such, understanding public interest and the accessibility of educational materials can provide insight into information-seeking behaviors and gaps in health literacy. This study used Google Trends (Google Limited Liability Corporation (LLC), Mountain View, CA) data to evaluate temporal patterns in public interest regarding ACL and MCL injuries and surgeries and to assess the readability of commonly accessed online educational materials, with the hypothesis that ACL injury and surgery-related terms would generate greater search interest and that the readability of articles for both ACL surgery and MCL surgery will exceed the American Medical Association's (AMA)-recommended sixth-grade reading level.

Methods: Google Trends was used to extract relative search volume (RSV) data in the United States from January 2004 through December 2025 for ACL- and MCL-related injury and surgical search terms. Injury terms (e.g., “ACL tear,” “MCL tear”) and surgical terms (e.g., “ACL reconstruction,” “MCL surgery”) were queried. Monthly and seasonal variations in RSV were evaluated for ACL and MCL surgery terms. Readability analysis was performed on the first 25 text-based Google search results for “ACL surgery” and “MCL surgery” using Flesch Reading Ease and Flesch-Kincaid Grade Level metrics.

Results: Among injury-related terms, “ACL tear” demonstrated the highest mean RSV compared to the lowest searched term (p < 0.001). Both “ACL tear” and “MCL tear” showed significant increases in RSV from 2004 to 2025 (p < 0.001). Among surgical terms, “ACL surgery” had the highest mean RSV overall, while “MCL surgery” was the most frequently searched MCL-related surgical term. RSV for both ACL and MCL surgery increased significantly over time. No significant differences in RSV were measured across seasons or months for either ACL surgery or MCL surgery. On readability analysis, the mean Flesch-Kincaid Grade Level was 10.4 for ACL surgery materials and 10.1 for MCL surgery materials, exceeding AMA recommendations. Only 4% of ACL surgery articles and 0% of MCL surgery articles met the recommended sixth-grade reading level.

Conclusion: Public interest in ACL and MCL injuries, tears, and surgical treatments has substantially increased in the past two decades, with ACL injury and surgery-related terms generating greater search interest than MCL injury and surgery-related terms, respectively. Patients preferentially searched for “tear” terminology rather than “injury” terminology, and “surgery” was the most common surgical search term. Despite growing public interest in ACL and MCL information, the readability of online educational materials remains significantly above recommended levels, possibly limiting patient comprehension. Future efforts should focus on improving the accessibility and readability of online orthopedic educational resources.

## Introduction

Musculoskeletal ligament injuries of the knee are of significant clinical concern, especially in physically active populations, since functional stability is key for daily activities [1]. Specifically, among these injuries, medial collateral ligament (MCL) and anterior cruciate ligament (ACL) injuries are commonly encountered. However, they have differences in surgical management and education resources. ACL surgeries are inherently popular for their functional importance and overall injury rate in sports requiring cutting, landing, and pivoting [2]. On the other hand, MCL is connected to the medial meniscus, which makes it an even bigger target for injury when ruptured and is most common in sports that require rotations of the knee joint [3]. Nevertheless, the injury rate is not that high compared to the ACL. With the growing utilization of patient-reported outcomes, there is an increasing need for health literacy to fulfill patient satisfaction [4].

In conjunction with the increasing reliance on digital resources for health concerns, patients are shifting to online platforms to further their understanding of diagnosis and treatment options. This emphasizes the importance of readability and clarity in educational materials, especially for complex orthopedic search terms such as “ACL surgery” and “MCL surgery.” The American Medical Association (AMA) recommends that online educational materials should be written at an elementary school reading level, particularly a sixth-grade reading level [5,6]. There is significant variability in medical information available online regarding academic level, as articles can rank as low as fifth-grade level, all the way to college reading level [7]. With such a wide range in reading level, patient accessibility for online educational material can plummet, leading to an overall decrease in patient education. With ACL injuries more common in the young, active population, particularly college-aged individuals, ease in patient accessibility becomes more of a concern [8]. Effective patient education in an orthopedic setting can provide not only a fulfillment of patient satisfaction but can result in a much more favorable preoperative and postoperative care [9,10].

Prior investigations evaluating public interest in orthopedic conditions such as ACL and MCL injuries and the readability of online educational materials have demonstrated a discrepancy between patient understanding and the medical complexity. Studies assessing the patient readability levels on online sources regarding ACL surgeries have shown that online patient education materials have lacked content that could empower patients to engage with their healthcare providers [11]. Within orthopedic educational patient sources, information regarding ACL tears has among the highest average grade reading level and lowest reading ease score, explaining the large discrepancy in patient understanding [12]. However, there is still considerable variability with information presentation, with reading levels reaching as low as fifth-grade level and as high as college level, leading to a substantial gap in information and greater emphasis on the impact from certain websites versus others [7].

To address these gaps, this study aimed to characterize temporal patterns in US Google Search interest for ACL and MCL injury and surgery-related terms by estimating and comparing relative search volume (RSV). Specifically, we sought to (1) compare mean RSVs among ACL and MCL injury terms and, separately, among ACL- and MCL-related surgery terms, (2) evaluate the variation in timing of search interest by month and season, and (3) assess the reliability of commonly accessed online patient education materials for ACL surgery versus MCL surgery using Flesch Reading Ease and Flesch-Kincaid Grade Level scores. We hypothesized that Google Trends data would demonstrate an increasing public search interest in ACL surgery and MCL surgery over time, with "ACL surgery" exhibiting the highest search interest among surgical terms, and that the readability of articles for both ACL surgery and MCL surgery found via Google search would exceed the AMA-recommended sixth-grade reading level.

## Materials and methods

Study design

A retrospective longitudinal study on public interest in ACL and MCL injuries and surgery was conducted from January 2004 to December 2025 using Google Trends. Google Trends is an online application that measures the RSV for any Google search term. The RSV is a standardized numerical value that ranges from 0 to 100 and represents interest in a search term within a specific timeframe.

Primary and secondary study outcomes

The primary study outcomes were the mean RSV for “ACL and MCL injury terms” and “ACL and MCL surgery terms” between 2004 and 2025. The secondary study outcomes were the mean RSV for “ACL surgery” and “MCL surgery” between the four seasons, winter, fall, spring, and summer, and the mean RSV for “ACL surgery” and “MCL surgery” from each calendar month. The secondary outcomes also included the mean Flesch Reading Ease score and the mean Flesch-Kincaid reading level for the first 25 articles found within a Google search inquiry for “ACL surgery” and “MCL surgery”.

Data collection

For the primary outcomes in this study, four search terms, including quotations defined as injury terms, were searched using Google Trends data from January 2004 to December 2025 solely in the United States with the “Search Term” option: “ACL injury,” “MCL injury,” “ACL tear,” and “MCL tear.” After acquiring the overall trend, the mean RSV for each search term was analyzed and compared to determine which search term had the most interest. Afterwards, the highest terms in the ACL and MCL categories were compared to determine which injury search term had the most overall interest within the specified time period. Moreover, six search terms, including quotations defined as surgical terms, were separately queried using Google Trends data restricted to the United States from January 2004 to December 2025 with the “Search Term” option: “ACL surgery”, “ACL repair”, “ACL reconstruction", “MCL surgery”, “MCL repair”, and “MCL reconstruction”. Injury-related and surgery-related search terms were queried and analyzed separately because they represent distinct categories of information-seeking behavior with different clinical contexts and research questions.

For the secondary outcomes of the study, to determine whether individual months impacted the mean RSV of surgical terms, the mean RSV for “ACL surgery” and “MCL surgery” from January to December (2004-2025) were compared between various months. Additionally, the mean RSV for “ACL surgery” and “MCL surgery” was compared between the four seasons (fall, winter, spring, summer). A meteorological approach was used where winter was classified as December, January, February; spring was classified as March, April, May; summer was June, July, August; and fall was September, October, November. 

To evaluate the reading level of online educational materials on ACL surgery and MCL, a Google search for “ACL surgery” and “MCL surgery” was performed in incognito mode on March 3rd, 2026, independently. One investigator extracted the main body text from each article after removing advertisements, hyperlinks, references, and other non-content elements. The first 25 text-based articles retrieved from the search results for each term were included for readability analysis. The readability analysis was performed by using the Flesch Reading Ease score and the Flesch-Kincaid reading level. The Flesch Reading Ease score and the Flesch-Kincaid reading level were analyzed by placing the text from the articles into Microsoft Word. Afterwards, the average Flesch Reading Ease score and the Flesch-Kincaid reading level were collected, and the proportion of articles meeting the AMA’s reading level standard was calculated.

Statistical analysis

Independent two-sample t-tests were used to analyze mean RSV between ACL- and MCL-related search terms within the injury-related and surgery-related Google Trends queries, which were analyzed separately. Welch's one-way analysis of variance (ANOVA) was used to compare mean RSV among search terms within the injury-related query and separately within the surgery-related query since the assumption of homogeneity of variance was not met. When significant, pairwise comparisons were performed using the Games-Howell post hoc test. Standard one-way ANOVA was used to compare mean RSV values across months and seasons from January 2004 to December 2025 because the assumption of homogeneity of variance was satisfied. Temporal trends for the primary injury-related (ACL tear and MCL tear) and surgery-related (ACL surgery and MCL surgery) search terms were evaluated using linear regression models with time (month) as a continuous predictor. Newey-West heteroskedasticity- and autocorrelation-consistent standard errors were used to account for autocorrelation in the monthly RSV data. Continuous variables were reported as means and standard deviations. Statistical significance was defined as p <0.05. Welch's ANOVA, Games-Howell post hoc testing, and standard ANOVA were performed using Microsoft Excel (Microsoft Corp., Redmond, WA, USA) with the Real Statistics Resource Pack, while the linear regression analysis with Newey-West standard errors was conducted in R (version 4.6.1; R Foundation for Statistical Computing, Vienna, Austria). Readability was assessed using the Flesch Reading Ease and Flesch Kincaid Grade Level statistics generated by the readability function in Microsoft Word Version 16.110.1 (Microsoft 365, Microsoft Corp., Redmond, WA, USA), upon which Microsoft Excel Version 16.110.2 was used to determine the average.

Inclusion and exclusion criteria

Data from search terms that had a non-zero mean RSV from Google Trends between January 2004 and December 2025 were included and eligible for analysis in the study. The exclusion criteria included countries outside of the United States and states that had zero mean RSV for the respective search terms throughout the entire study period. In addition, if articles consisted of only visuals or videos, they were excluded because Microsoft Word could not analyze the article without text. The inclusion and exclusion criteria used in this study are summarized in Table 1.

**Table 1:** Exclusion and Inclusion Criteria

Criterion Type	Criterion
Inclusion	Any Google Trends search terms with a non-zero mean relative search volume between January 2004 and December 2025.
Inclusion	Search data only originating from the United States.
Exclusion	Countries outside of the United States.
Exclusion	Search terms with a relative search volume of zero throughout the entire study period were excluded from analysis.
Exclusion	Articles consisting only of visuals, videos, or other non-text content that could not be analyzed using Microsoft Word readability software.

## Results

Comparison of search terms: Injury terms

Among the four injury terms, “ACL tear” had the highest mean RSV, followed by “ACL injury,” “MCL tear,” and “MCL injury” (31.07 vs. 14.54 vs. 8.64 vs. 5.02, respectively; Welch’s ANOVA p<0.001) (Table 2). Games-Howell post-hoc analysis demonstrated that “ACL tear” was significantly higher than all other injury-related search terms (p<0.001). Additionally, “MCL tear” showed a significantly higher mean RSV compared to “MCL injury” (8.64 vs. 5.02; mean difference: 3.61; 95% CI: 2.82-4.41; p<0.001) (Table 3).

**Table 2:** Mean Relative Search Volume for ACL and MCL Injury-Related Search Terms ACL, anterior cruciate ligament; MCL, medial collateral ligament.

Group	Count	Average	Variance	Welch’s F	df1	df2	p-Value
ACL injury	264	14.54	23.46	562.28	3	534.53	<0.0001
MCL injury	264	5.02	5.82	562.28	3	534.53	<0.0001
ACL tear	264	31.07	174.13	562.28	3	534.53	<0.0001
MCL tear	264	8.64	19.29	562.28	3	534.53	<0.0001

**Table 3:** Games-Howell Post Hoc Test ACL and MCL Injury Terms ACL, anterior cruciate ligament; MCL, medial collateral ligament.

Comparison	Mean Difference	Lower Bound	Upper Bound	p-Value
ACL tear - ACL injury	16.53	14.30	18.77	<0.0001
ACL tear - MCL tear	22.44	20.22	24.65	<0.0001
ACL tear - MCL injury	26.05	23.92	28.18	<0.0001
ACL injury - MCL tear	5.90	4.87	6.94	<0.0001
ACL injury - MCL injury	9.52	8.66	10.37	<0.0001
MCL tear - MCL injury	3.61	2.82	4.41	<0.0001

**Figure 1:**
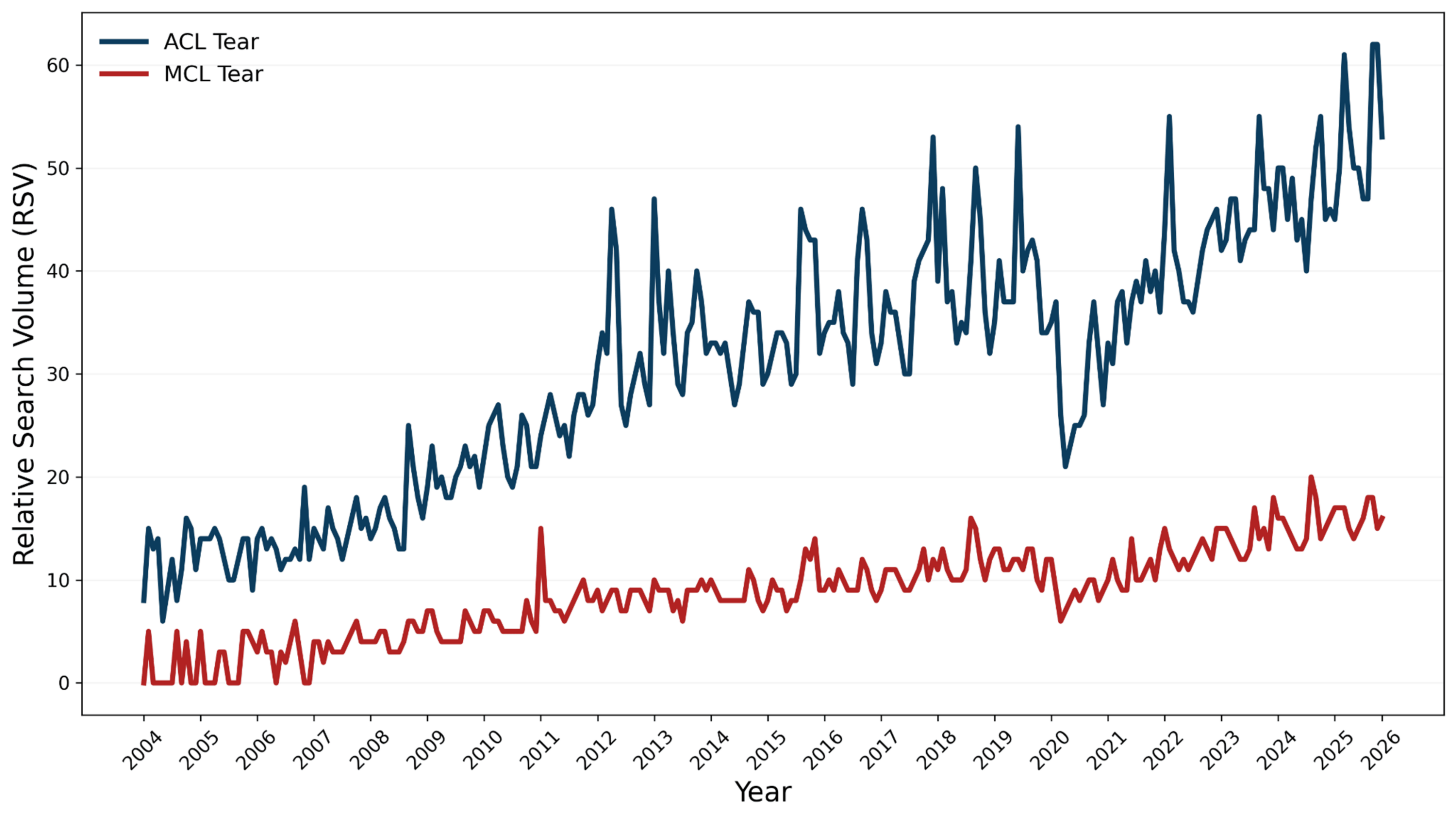
Relative Search Volume for ACL and MCL Tear in the United States, 2004-2025 RSV data were obtained from Google Trends for ACL and MCL tears. The blue line represents search interest for "ACL tear," and the red line represents search interest for "MCL tear". RSV, relative search volume; ACL, anterior cruciate ligament; MCL, medial collateral ligament.

Moreover, when comparing annual mean RSV values between 2004 and 2025, both “ACL tear” and “MCL tear” had risen significantly (2004 - 11.5 vs. 2005 - 56.8, p<0.05; 2004 - 1.2 vs. 2025 - 16.2, p<0.05, respectively) (Figure 1). After conducting linear regression with Newey-West standard errors to account for autocorrelation, both search terms showed significant positive temporal trends from 2004 to 2025. “ACL tear” demonstrated an increase of 0.150 RSV per month (β=0.150; Newey-West 95% CI: 0.123-0.178; p<0.001; R²=0.758). While “MCL tear” demonstrated an increase of 0.052 RSV per month (β=0.052; 95% CI: 0.047-0.057; p<0.001; R²=0.816) (Table 4).

**Table 4:** Linear Regression Analysis With Newey-West Standard Errors RSV, relative search volume; ACL, anterior cruciate ligament; MCL, medial collateral ligament.

Search Term	β Coefficient (RSV/month)	Newey-West SE	Newey-West 95% CI	p-Value	R²
ACL surgery	0.210	0.012	0.187-0.234	<0.001	0.861
MCL surgery	0.200	0.011	0.180-0.221	<0.001	0.645
ACL tear	0.150	0.014	0.123-0.178	<0.001	0.758
MCL tear	0.052	0.003	0.047-0.057	<0.001	0.816

Comparison of search terms: Surgery terms

Among the six surgical terms, “ACL surgery” had the highest mean RSV (52.95), followed by “MCL surgery” (25.58), “ACL reconstruction” (17.30), “ACL repair” (8.51), “MCL repair” (7.96), and “MCL reconstruction” (1.91) (Table 5). Games-Howell post-hoc testing demonstrated that “ACL surgery” was significantly higher than all other surgical search terms, including “MCL surgery” (mean difference: 27.38; 95% CI: 22.86-31.89; p<0.001) (Table 6). Among MCL terms, “MCL surgery” was significantly higher than both “MCL repair” and “MCL reconstruction” (p<0.001).

**Table 5:** Mean RSV of ACL and MCL Surgery-Related Terms RSV, relative search volume; ACL, anterior cruciate ligament; MCL, medial collateral ligament.

Search Term	Count	Mean RSV	Variance	Welch’s F	df1	df2	p-Value
ACL surgery	264	52.95	299.40	783.30	5	693.94	<0.0001
ACL repair	264	8.51	4.56	783.30	5	693.94	<0.0001
ACL reconstruction	264	17.30	12.89	783.30	5	693.94	<0.0001
MCL surgery	264	25.58	363.29	783.30	5	693.94	<0.0001
MCL repair	264	7.96	77.59	783.30	5	693.94	<0.0001
MCL reconstruction	264	1.91	19.36	783.30	5	693.94	<0.0001

**Table 6:** Games-Howell Post Hoc Test ACL and MCL Surgery Terms ACL, anterior cruciate ligament; MCL, medial collateral ligament.

Comparison	Mean Difference	Lower Bound	Upper Bound	p-Value
ACL surgery - MCL surgery	27.38	22.86	31.89	<0.001
ACL surgery - ACL reconstruction	35.65	32.53	38.77	<0.001
ACL surgery - ACL repair	44.44	41.36	47.52	<0.001
ACL surgery - MCL repair	44.99	41.57	48.41	<0.001
ACL surgery - MCL reconstruction	51.05	47.90	54.20	<0.001
MCL surgery - ACL reconstruction	8.28	4.85	11.70	<0.001
MCL surgery - ACL repair	17.07	13.68	20.46	<0.001
MCL surgery - MCL repair	17.62	13.92	21.32	<0.001
MCL surgery - MCL reconstruction	23.67	20.22	27.13	<0.001
ACL reconstruction - ACL repair	8.79	8.06	9.53	<0.001
ACL reconstruction - MCL repair	9.34	7.66	11.02	<0.001
ACL reconstruction - MCL reconstruction	15.40	14.40	16.39	<0.001
ACL repair - MCL repair	0.55	-1.05	2.15	0.92
ACL repair - MCL reconstruction	6.61	5.74	7.47	<0.001
MCL repair - MCL reconstruction	6.06	4.32	7.79	<0.001

**Figure 2:**
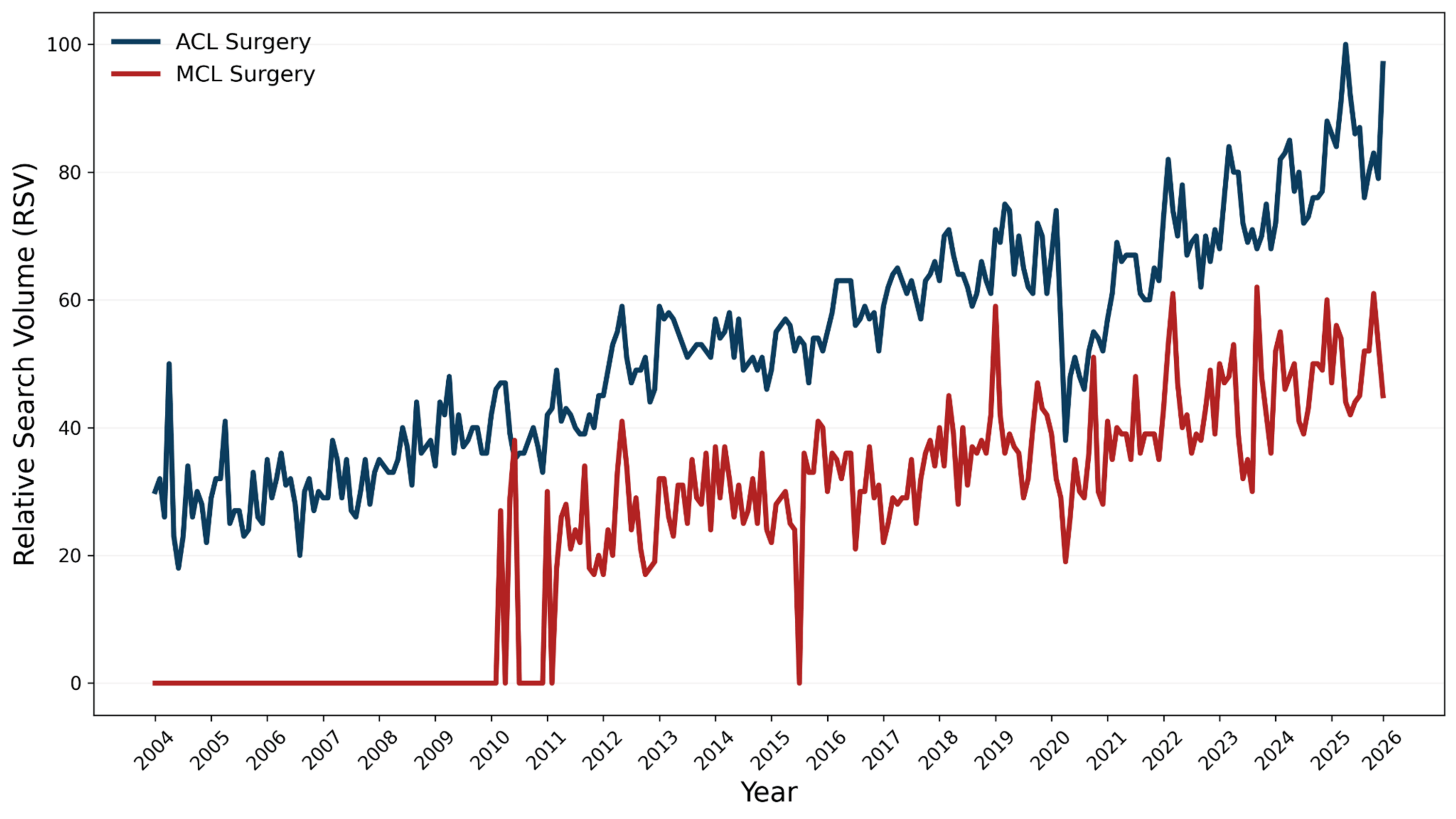
RSV for ACL and MCL Surgery in the United States, 2004-2025 RSV data were obtained from Google Trends for ACL and MCL surgery. The blue line represents search interest for "ACL surgery," and the red line represents search interest for "MCL surgery." RSV, relative search volume; ACL, anterior cruciate ligament; MCL, medial collateral ligament.

Moreover, both ACL surgery and MCL surgery mean RSV significantly increased from 2004 to 2025 (2004 - 28.5, 2025 - 86.75, and 2004 - 0, 2025 - 21.92, p<0.05) (Figure 2). After conducting linear regression with Newey-West standard errors to account for autocorrelation, both surgical search terms showed significant positive temporal trends from 2004 to 2025. “ACL surgery” demonstrated an increase of 0.210 RSV per month (β=0.210; Newey-West 95% CI: 0.187-0.234; p<0.001; R²=0.861), while “MCL surgery” demonstrated an increase of 0.200 RSV per month (β=0.200; Newey-West 95% CI: 0.180-0.221; p<0.001; R²=0.645) (Table 4).

Impact of month and season on ACL and MCL surgery

Statistical analysis of the mean RSV for “MCL surgery” for winter, fall, spring, and summer from 2004 to 2025 indicated no significant differences (winter, 25.4; spring, 25.8; summer, 23.3; fall, 27.7; p=0.625) (Table 7). Statistical analysis of the mean RSV for “MCL surgery” from January to December from 2004 to 2025 showed no significant differences (January, 28.7; February, 24; March, 26.4; April, 26.2; May, 24.8; June, 26.9; July, 20.3; August, 22.8; September, 25.6; October, 30.5; November, 27.1; December, 23.6; p=0.938) (Table 8). 

**Table 7:** Mean Relative Search Volume for "MCL Surgery" by Season MCL, medial collateral ligament.

Group	Count	Sum	Mean Relative Search Volume	Variance	F	p-Value
Winter	66	1679	25.44	406.56	0.586	0.624
Spring	66	1702	25.79	321.80	0.586	0.624
Summer	66	1541	23.35	277.55	0.586	0.624
Fall	66	1831	27.74	454.16	0.586	0.624

**Table 8:** Mean Relative Search Volume for "MCL Surgery" by Month MCL, medial collateral ligament.

Month	Count	Sum	Mean Relative Search Volume	Variance	F	p-Value
Jan	22	632	28.73	470.49	0.438	0.938
Feb	22	528	24.00	420.10	0.438	0.938
Mar	22	580	26.36	377.77	0.438	0.938
Apr	22	576	26.18	336.35	0.438	0.938
May	22	546	24.82	280.44	0.438	0.938
Jun	22	592	26.91	254.56	0.438	0.938
Jul	22	447	20.32	288.51	0.438	0.938
Aug	22	502	22.82	292.82	0.438	0.938
Sep	22	564	25.64	391.48	0.438	0.938
Oct	22	671	30.50	640.93	0.438	0.938
Nov	22	596	27.09	360.28	0.438	0.938
Dec	22	519	23.59	350.73	0.438	0.938

Statistical analysis of the mean RSV for “ACL surgery” for winter, fall, spring, and summer from 2004 to 2025 indicated no significant differences (winter, 53.3; spring, 56; summer, 51; fall, 51.6; p=0.348) (Table 9). Statistical analysis of the mean RSV for “ACL surgery” from January to December from 2004 to 2025 showed no significant differences (January, 52.6; February, 55.5; March, 56.9; April, 57.5; May, 53.5; June, 53; July, 50.6; August, 49.4; September, 50.3; October, 53; November, 51.4; December, 51.6; p=0.917) (Table 10).

**Table 9:** Mean Relative Search Volume for "ACL Surgery" by Season ACL, anterior cruciate ligament.

Group	Count	Sum	Mean Relative Search Volume	Variance	F	p-Value
Winter	66	3515	53.26	317.92	1.105	0.348
Spring	66	3695	55.98	333.49	1.105	0.348
Summer	66	3365	50.98	291.18	1.105	0.348
Fall	66	3405	51.59	253.60	1.105	0.348

**Table 10:** Mean Relative Search Volume for "ACL Surgery" by Month ACL, anterior cruciate ligament.

Group	Count	Sum	Mean Relative Search Volume	Variance	F	p-Value
Jan	22	1157	52.59	276.73	0.476	0.917
Feb	22	1222	55.55	326.07	0.476	0.917
Mar	22	1251	56.86	340.89	0.476	0.917
Apr	22	1266	57.55	309.69	0.476	0.917
May	22	1178	53.55	372.07	0.476	0.917
Jun	22	1165	52.95	312.90	0.476	0.917
Jul	22	1114	50.64	303.10	0.476	0.917
Aug	22	1086	49.36	278.34	0.476	0.917
Sep	22	1107	50.32	237.66	0.476	0.917
Oct	22	1167	53.05	246.14	0.476	0.917
Nov	22	1131	51.41	297.21	0.476	0.917
Dec	22	1136	51.64	372.53	0.476	0.917

Analysis of reading level and ease

From the 25 articles analyzed from a Google search for “ACL surgery”, the mean Flesch Kincaid Grade reading level was 10.4, and the mean Flesch Reading Ease score was 49.192. Among the 25 articles analyzed for “MCL surgery,” the mean Flesch Kincaid Grade reading level was 10.1, and the mean Flesch Reading Ease score was 53.304. In addition, only one out of the 25 articles (4%) analyzed for “ACL surgery” patient education was at the AMA-recommended sixth-grade reading level. Analysis of the “MCL surgery” term for patient education showed that none of the 25 articles (0%) met the AMA reading level recommendation.

## Discussion

Between 2004 and 2025, RSV values for ACL and MCL injuries have steadily risen. A key reason that may explain this growth is that the age at which children undergo sports specialization is becoming increasingly younger [13]. Intense training in one particular sport has been shown to increase the likelihood of injuries in those athletes [14]. These trends may underlie an increase in the incidence of ACL and MCL injuries, where a greater number of injured athletes use internet searches to self-evaluate or understand their diagnosis. Moreover, there was a significantly higher RSV for an ACL or MCL “tear” search term than the respective ACL/MCL “injury.” This could be due to the relative negative framing of a tear versus the relative neutral framing of an injury. Patients and physicians are shown to be equally susceptible to framing biases in clinical contexts [15]; although not testable by the present data, this may partially explain the apparent increase in search interest for “tears” rather than “injuries.” However, there was no statistically significant variation in RSVs for “ACL surgery” or “MCL surgery” across different seasons or months. Insurance-related delays have been documented in orthopedic procedures [16] and may introduce significant lag times between injury and surgical scheduling. Therefore, fluctuations in injury incidence may attenuate the seasonality of injury patterns into a more uniform level of interest among search terms regarding anterior cruciate ligament reconstruction (ACLR) and medial collateral ligament reconstruction (MCLR). However, this remains a hypothesis that the present data cannot directly test.

Among all six included surgical terms, “ACL surgery” had the highest mean RSV overall, while the highest MCL term RSV was “MCL surgery.” The RSV for ACL/MCL “reconstruction” was the lowest within each group of ACL or MCL surgical terms. This disparity likely stems from a consumer-professional vocabulary gap [17]. In the acute phase of injury, patients mirror early-stage researchers who prioritize broad searches driven by colloquial terminology like “surgery.” The technical term “reconstruction” may only be adopted by the patient after formal clinical consultation or if the patient is already well-educated on surgical terminology. Moreover, due to the high reading level of most orthopedic materials, patients may outright filter out the word “reconstruction” or replace it with “surgery” when they encounter it [18]. This may constitute the persistence of “surgery” as the predominant term when patients are referring to ACLR and MCLR even after clinical consultation.

RSV values for a given ACL injury term were also noted to be higher than for the corresponding MCL injury term. This may be attributed to multifaceted reasons such as clinical impact, recovery burden, and disruption of athletic activity associated with ACL compared with MCL injuries. A key aspect of this is that ACL injuries are far more likely to require surgery than MCL injuries [19]. This may be partially explained by differences in typical treatment approaches, as ACL tears more commonly have a surgical recommendation as first-line treatment, while MCL injuries’ medical intervention is most often non-surgical and early rehabilitation [20,21]. Therefore, increased treatment complexity and recovery considerations may contribute to greater public search interest surrounding ACL-related injuries compared with MCL-related injuries. ACL tears are also shown to carry risks of osteoarthritis, in which patients may experience recurrence of knee pain after an ACL tear and search for “ACL” terms regarding reinjury or recovery [22].

Despite the rising number of searches regarding ACL and MCL injuries/tears, published research articles often exceed patients’ reading levels. The AMA recommends that patient articles should be written at a sixth-grade level for patient comprehension [23]. However, the Flesch Kincaid grade for these articles was, on average, significantly higher than the recommendations by the AMA for patient comprehension; the mean reading level for patient education articles on MCLR was 10.124, and ACLR was 10.428, corresponding to a reading level between the 10th and 11th grade [24]. Patients often lack proper understanding of medical terminologies due to language barriers and cultural differences, combined with the shortage and time constraints of medical staff to educate patients; the barrier between research articles and patient comprehension gap widens [25]. This suggests that patient educational materials that exceed recommended readability levels may represent a barrier to accessible health information and overall clarity. Moreover, zero (0%) patient resources on MCLR had a Flesch Kincaid grade equivalent to the sixth-grade level, and only 1/25 (4%) of patient resources on ACLR had a Flesch Kincaid grade equivalent to the sixth-grade level. The gap between the complexity of patient materials and the AMA recommendations creates a barrier for patients that hampers the accessibility of patient education on ACLR and MCLR.

While professional surgical organizations often produce materials for patient education on procedures like ACLR and MCLR, such origins of patient materials are from expert clinical communication jargon and structure [26]. Unfortunately, this structure and medical language are often left undersimplified, which may further explain the persistent complexity when literature is repurposed for materials for patient education [27]. While the source of patient material may be difficult to simplify, dedicating time to translating medical languages to simpler terminologies and altering sentence structures is paramount to increasing the ease for patients to absorb information from disseminated material. Increasing patient readability of ACLR and MCLR materials may contribute to increasing patients’ knowledge, allow for self-advocacy, and ultimately improve outcomes in medical care.

Limitations of this study include the inability of Google Trends to give causal data on what prompted an individual to search a given term. Thus, it is unclear whether the RSV of a search term was higher during a given timeframe due to an increase in the prevalence of that injury or if it was due to media coverage of a high-profile sporting injury. Additionally, because all search terms could not be analyzed simultaneously within a single Google Trends query, injury-related and surgery-related terms were evaluated separately, limiting direct comparisons between these categories. Furthermore, Google search results may vary based on factors such as time, device, location, and browser settings, which may impact reproducibility. The initial Google search results for the readability analysis included a heterogeneous mix of patient education resources, commercial clinic websites, and other online materials, which may influence overall readability estimates. Additionally, the exclusion of videos, images, and other multimedia resources omits an increasingly common method through which patients access health information. Future studies may benefit from evaluating a larger sample of articles, stratifying resources by source type, and incorporating additional readability metrics. Finally, the findings may have limited generalizability because the analysis was restricted to English-language Google search results within the United States. For future studies, it may be beneficial to replicate the study with the addition of geographical variations, including but not limited to the differences on a state-to-state basis, to determine regions lacking sufficient medical education. Moreover, given the limited understanding of the underlying causal relationship of RSV, further investigation to explain the factors driving increased awareness of ACLR and MCLR and search prevalence is necessary.

## Conclusions

Public interest in ACL and MCL injuries, tears, and surgeries has all increased over time; however, ACL injury and surgery topics have garnered significantly more attention. Moreover, interest in MCL surgeries showed no significant monthly fluctuations. Individuals were most likely to search “ACL tear” or “MCL tear” when attempting to gather information on an ACL or MCL injury, respectively. However, despite the increase in public interest in ACL and MCL injuries/tears/surgeries, the articles most likely to be accessed by individuals are, on average, beyond the AMA recommendations for healthcare information to be at a sixth-grade reading level or lower. Future endeavors to increase readability should be done to ensure effective patient understandability when educational materials are accessed.
